# 1-[(6-Chloro-3-pyrid­yl)meth­yl]-5-eth­oxy-8-nitro-1,2,3,5,6,7-hexa­hydro­imidazo[1,2-*a*]pyridine

**DOI:** 10.1107/S1600536809037660

**Published:** 2009-09-26

**Authors:** Zhongzhen Tian, Dongmei Li, Zhong Li

**Affiliations:** a Institute of Chemistry and Chemical Engineering, University of Jinan, 106 Jiwei Road, Jinan 250022, People’s Republic of China; b Shanghai Key Laboratory of Chemical Biology, School of Pharmacy, East China University of Science and Technology, Shanghai 200237, People’s Republic of China

## Abstract

In the title compound, C_15_H_19_ClN_4_O_3_, an active agrochemical possessing insecticidal activity, the dihedral angle between the mean planes passing through the pyridine ring and the five-membered ring is 87.3 (2)°. The fused pyridine ring adopts a twisted sofa conformation. The mol­ecular structure features close intra­molecular C—H⋯N and C—H⋯O hydrogen bonding.

## Related literature

For related literature, see: Kagabu *et al.* (2002 ▶); Moriya *et al.* (1992 ▶); Tian *et al.* (2007 ▶); Tokumitsu (1990 ▶).
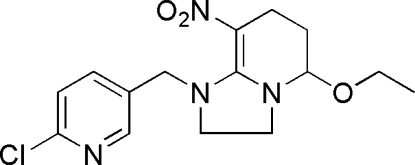


## Experimental

### 

#### Crystal data

C_15_H_19_ClN_4_O_3_
*M* *_r_* = 338.79
Monoclinic, 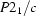

*a* = 17.021 (3) Å
*b* = 5.5737 (8) Å
*c* = 18.334 (3) Å
β = 112.097 (3)°
*V* = 1611.6 (4) Å^3^
*Z* = 4
Mo *K*α radiation
μ = 0.26 mm^−1^
*T* = 290 K
0.50 × 0.24 × 0.12 mm

#### Data collection

Bruker SMART CCD area-detector diffractometer
Absorption correction: multi-scan (*SADABS*; Bruker, 1997 ▶) *T*_min_ = 0.922, *T*_max_ = 0.969
8970 measured reflections
3486 independent reflections
1870 reflections with *I* > 2σ(*I*)
*R*_int_ = 0.082

#### Refinement

*R*[*F* ^2^ > 2σ(*F* ^2^)] = 0.049
*wR*(*F* ^2^) = 0.135
*S* = 0.82
3486 reflections
210 parameters
H-atom parameters constrained
Δρ_max_ = 0.32 e Å^−3^
Δρ_min_ = −0.24 e Å^−3^

### 

Data collection: *SMART* (Bruker, 1997 ▶); cell refinement: *SAINT* (Bruker, 1997 ▶); data reduction: *SAINT*; program(s) used to solve structure: *SHELXS97* (Sheldrick, 2008 ▶); program(s) used to refine structure: *SHELXL97* (Sheldrick, 2008 ▶); molecular graphics: *ORTEP-3* (Farrugia, 1997 ▶); software used to prepare material for publication: *SHELXTL* (Sheldrick, 2008 ▶).

## Supplementary Material

Crystal structure: contains datablocks global, I. DOI: 10.1107/S1600536809037660/bx2230sup1.cif

Structure factors: contains datablocks I. DOI: 10.1107/S1600536809037660/bx2230Isup2.hkl

Additional supplementary materials:  crystallographic information; 3D view; checkCIF report

## Figures and Tables

**Table 1:** Hydrogen-bond geometry (Å, °)

*D*—H⋯*A*	*D*—H	H⋯*A*	*D*⋯*A*	*D*—H⋯*A*
C11—H11*B*⋯O3	0.97	2.35	2.803 (3)	108
C13—H13⋯N1	0.93	2.54	2.891 (3)	103

**Fig. 1.**
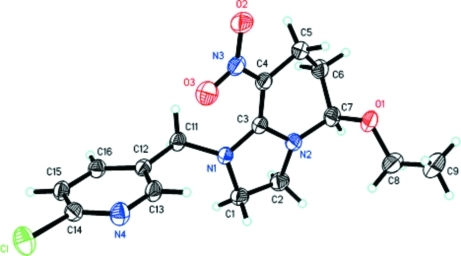
The molecular structure of (a) with atom numbering scheme. Displacement ellipsoids are drawn at the 30% probability level. The H atoms are shown as circles of arbitrary size and intramolecular hydrogen bonds are indicated by dotted lines.

## supplementary crystallographic information

### Comment

Since the debut of Imidacloprid in  the 1990 decade  (Moriya *et al.*, 1992), neonicotinoid insecticides have become rapidly an important chemical class of insecticides. Nitromethylene compounds (Kagabu *et al.*, 2002) exhibited remarkably higher biological activity but poor photostability compared with Imidacloprid. Our synthetic interest was introducing dicyclic ring into the lead structure to improve its photostability and synthesizing a series of new compounds, in which compound (I) exhibited good insecticidal activities against pea aphids and was slightly weaker than that of imidacloprid (Tian *et al.*, 2007).The dihedral angle between the mean planes passing through the pyridine ring and the five membered ring is 87.3 (2)°. The six membered N2/C3/C4/C5/C6/C7 ring adopts a twist sofa conformation. The title compound C_15_H_19_ClN_4_O_3_ , is an active agrochemical possessing insecticidal activity. The dihedral angle between the mean planes passing through the pyridine ring and the five membered ring is 87.3 (2)°. The six membered N2/C3/C4/C5/C6/C7 ring adopts a twist sofa conformation.The molecular structure is stabilized by intramolecular C— H··· N and C— H··· O hydrogen bond, Table 1.

### Experimental

The synthesis of the title compoud was following the reported method by Tokumitsu, 1990. Single crystals suitable for X-ray analysis were obtained by slow evaporation of the solution of dichloromethane and petroleum ether of the title compound. m.p. 399.8–400.8 K.

### Refinement

H atoms were positioned geometrically and included in the refinement in the riding-model approximation, with C—H = 0.93 Å and *U*_iso_(H) = 1.2 *U*_eq_(C) for pyridine H atoms, C—H = 0.97 Å and *U*_iso_(H) = 1.2 *U*_eq_(C) for the methylene H atoms, C—H = 0.96 Å and *U*_iso_(H) = 1.5 *U*_eq_(C) for the methyl H atoms, C—H = 0.98 Å and *U*_iso_(H) = 1.2 *U*_eq_(C) for the methenyl H atoms

### Crystal data

**Table:**

C_15_H_19_ClN_4_O_3_	*F*(000) = 712
*M**_r_* = 338.79	*D*_x_ = 1.396 Mg m^−^^3^
Monoclinic, *P*2_1_/*c*	Mo *K*α radiation, λ = 0.71073 Å
Hall symbol: -P 2ybc	Cell parameters from 1727 reflections
*a* = 17.021 (3) Å	θ = 4.8–47.4°
*b* = 5.5737 (8) Å	µ = 0.26 mm^−^^1^
*c* = 18.334 (3) Å	*T* = 290 K
β = 112.097 (3)°	Prismatic, colourless
*V* = 1611.6 (4) Å^3^	0.50 × 0.24 × 0.12 mm
*Z* = 4	

### Data collection

**Table:**

Bruker SMART CCD area-detector diffractometer	3486 independent reflections
Radiation source: fine-focus sealed tube	1870 reflections with *I* > 2σ(*I*)
graphite	*R*_int_ = 0.082
φ and ω scans	θ_max_ = 27.0°, θ_min_ = 1.3°
Absorption correction: multi-scan (*SADABS*; Bruker, 1997)	*h* = −21→21
*T*_min_ = 0.922, *T*_max_ = 0.969	*k* = −7→7
8970 measured reflections	*l* = −23→13

### Refinement

**Table:**

Refinement on *F*^2^	Secondary atom site location: difference Fourier map
Least-squares matrix: full	Hydrogen site location: inferred from neighbouring sites
*R*[*F*^2^ > 2σ(*F*^2^)] = 0.049	H-atom parameters constrained
*wR*(*F*^2^) = 0.135	*w* = 1/[σ^2^(*F*_o_^2^) + (0.069*P*)^2^] where *P* = (*F*_o_^2^ + 2*F*_c_^2^)/3
*S* = 0.82	(Δ/σ)_max_ = 0.044
3486 reflections	Δρ_max_ = 0.32 e Å^−^^3^
210 parameters	Δρ_min_ = −0.24 e Å^−^^3^
0 restraints	Extinction correction: *SHELXL97* (Sheldrick, 2008), Fc^*^=kFc[1+0.001xFc^2^λ^3^/sin(2θ)]^-1/4^
Primary atom site location: structure-invariant direct methods	Extinction coefficient: 0.020 (2)

### Special details

**Table:**

Geometry. All e.s.d.'s (except the e.s.d. in the dihedral angle between two l.s. planes) are estimated using the full covariance matrix. The cell e.s.d.'s are taken into account individually in the estimation of e.s.d.'s in distances, angles and torsion angles; correlations between e.s.d.'s in cell parameters are only used when they are defined by crystal symmetry. An approximate (isotropic) treatment of cell e.s.d.'s is used for estimating e.s.d.'s involving l.s. planes.
Refinement. Refinement of *F*^2^ against ALL reflections. The weighted *R*-factor *wR* and goodness of fit *S* are based on *F*^2^, conventional *R*-factors *R* are based on *F*, with *F* set to zero for negative *F*^2^. The threshold expression of *F*^2^ > σ(*F*^2^) is used only for calculating *R*-factors(gt) *etc*. and is not relevant to the choice of reflections for refinement. *R*-factors based on *F*^2^ are statistically about twice as large as those based on *F*, and *R*- factors based on ALL data will be even larger.

### Fractional atomic coordinates and isotropic or equivalent isotropic displacement parameters (Å^2^)

**Table:**

	*x*	*y*	*z*	*U*_iso_*/*U*_eq_	
Cl	1.20916 (4)	0.34129 (13)	0.43476 (5)	0.0742 (3)	
O1	0.58663 (11)	0.9036 (4)	0.35790 (10)	0.0669 (5)	
O2	0.69588 (12)	0.4707 (4)	0.16844 (11)	0.0739 (6)	
O3	0.81680 (12)	0.5033 (3)	0.26680 (11)	0.0653 (5)	
N1	0.84352 (11)	0.9213 (3)	0.35853 (10)	0.0428 (5)	
N2	0.71934 (11)	1.0384 (4)	0.35902 (11)	0.0484 (5)	
N3	0.74299 (13)	0.5777 (4)	0.22900 (12)	0.0517 (5)	
N4	1.05820 (12)	0.4680 (4)	0.42526 (12)	0.0542 (5)	
C1	0.86309 (14)	1.0659 (5)	0.43064 (14)	0.0528 (6)	
H1A	0.9076	1.1814	0.4361	0.063*	
H1B	0.8806	0.9645	0.4770	0.063*	
C2	0.77991 (15)	1.1909 (5)	0.41832 (15)	0.0594 (7)	
H2A	0.7687	1.1946	0.4664	0.071*	
H2B	0.7793	1.3533	0.3991	0.071*	
C3	0.75815 (13)	0.9028 (4)	0.32102 (13)	0.0406 (5)	
C4	0.71043 (14)	0.7723 (4)	0.25471 (13)	0.0451 (6)	
C5	0.61813 (14)	0.8220 (5)	0.21354 (14)	0.0582 (7)	
H5A	0.5857	0.6921	0.2236	0.070*	
H5B	0.6045	0.8293	0.1572	0.070*	
C6	0.59349 (15)	1.0563 (5)	0.24097 (15)	0.0602 (7)	
H6A	0.5322	1.0689	0.2210	0.072*	
H6B	0.6150	1.1890	0.2197	0.072*	
C7	0.62807 (15)	1.0737 (5)	0.32935 (15)	0.0552 (7)	
H7	0.6161	1.2340	0.3445	0.066*	
C8	0.59553 (18)	0.9382 (6)	0.43754 (16)	0.0753 (9)	
H8A	0.5732	1.0941	0.4433	0.090*	
H8B	0.6551	0.9334	0.4715	0.090*	
C9	0.5492 (2)	0.7483 (7)	0.46073 (19)	0.1023 (12)	
H9A	0.4896	0.7620	0.4299	0.123*	
H9B	0.5587	0.7652	0.5155	0.123*	
H9C	0.5691	0.5940	0.4519	0.123*	
C11	0.89956 (13)	0.9436 (4)	0.31525 (13)	0.0455 (6)	
H11A	0.9131	1.1118	0.3130	0.055*	
H11B	0.8695	0.8891	0.2617	0.055*	
C12	0.98050 (13)	0.8050 (4)	0.35017 (12)	0.0393 (5)	
C13	0.98935 (14)	0.6091 (4)	0.39838 (13)	0.0490 (6)	
H13	0.9443	0.5719	0.4134	0.059*	
C14	1.12093 (14)	0.5302 (4)	0.40378 (13)	0.0454 (6)	
C15	1.12235 (14)	0.7269 (4)	0.35956 (13)	0.0465 (6)	
H15	1.1699	0.7651	0.3486	0.056*	
C16	1.05008 (14)	0.8655 (4)	0.33212 (13)	0.0462 (6)	
H16	1.0480	1.0000	0.3014	0.055*	

### Atomic displacement parameters (Å^2^)

**Table:**

	*U*^11^	*U*^22^	*U*^33^	*U*^12^	*U*^13^	*U*^23^
Cl	0.0596 (4)	0.0681 (5)	0.0967 (6)	0.0181 (3)	0.0313 (4)	0.0041 (4)
O1	0.0668 (11)	0.0879 (15)	0.0610 (12)	−0.0114 (10)	0.0413 (10)	−0.0097 (10)
O2	0.0778 (12)	0.0823 (14)	0.0702 (13)	−0.0259 (11)	0.0374 (11)	−0.0376 (11)
O3	0.0691 (12)	0.0575 (12)	0.0737 (13)	0.0125 (9)	0.0320 (10)	−0.0075 (9)
N1	0.0411 (11)	0.0494 (12)	0.0425 (11)	−0.0023 (8)	0.0210 (9)	−0.0026 (9)
N2	0.0439 (11)	0.0575 (13)	0.0477 (12)	0.0013 (9)	0.0216 (9)	−0.0130 (10)
N3	0.0583 (13)	0.0558 (13)	0.0503 (13)	−0.0090 (11)	0.0311 (11)	−0.0095 (11)
N4	0.0553 (12)	0.0513 (13)	0.0624 (14)	0.0059 (10)	0.0293 (11)	0.0106 (10)
C1	0.0529 (14)	0.0594 (16)	0.0473 (14)	−0.0102 (12)	0.0201 (12)	−0.0093 (12)
C2	0.0641 (16)	0.0623 (18)	0.0556 (16)	−0.0076 (13)	0.0269 (13)	−0.0186 (13)
C3	0.0447 (13)	0.0413 (13)	0.0407 (13)	0.0016 (10)	0.0216 (11)	0.0027 (10)
C4	0.0475 (13)	0.0493 (15)	0.0436 (14)	−0.0033 (11)	0.0231 (11)	−0.0055 (11)
C5	0.0476 (14)	0.081 (2)	0.0484 (15)	−0.0038 (13)	0.0204 (12)	−0.0060 (13)
C6	0.0466 (14)	0.082 (2)	0.0545 (16)	0.0109 (13)	0.0218 (12)	0.0084 (14)
C7	0.0500 (14)	0.0604 (17)	0.0626 (17)	0.0079 (12)	0.0297 (13)	−0.0033 (13)
C8	0.0739 (19)	0.104 (3)	0.0543 (18)	0.0098 (17)	0.0314 (15)	0.0030 (17)
C9	0.111 (3)	0.143 (3)	0.071 (2)	−0.020 (2)	0.055 (2)	0.006 (2)
C11	0.0480 (13)	0.0474 (14)	0.0479 (14)	−0.0009 (11)	0.0257 (11)	0.0059 (11)
C12	0.0426 (12)	0.0410 (13)	0.0379 (12)	−0.0033 (10)	0.0193 (10)	−0.0017 (10)
C13	0.0483 (14)	0.0533 (16)	0.0545 (15)	−0.0001 (11)	0.0299 (12)	0.0080 (12)
C14	0.0433 (13)	0.0477 (15)	0.0453 (14)	0.0016 (11)	0.0167 (11)	−0.0066 (11)
C15	0.0435 (13)	0.0538 (15)	0.0494 (14)	−0.0068 (11)	0.0257 (11)	−0.0048 (12)
C16	0.0488 (14)	0.0493 (14)	0.0451 (14)	−0.0073 (11)	0.0230 (11)	0.0045 (11)

### Geometric parameters (Å, °)

**Table:**

Cl—C14	1.745 (2)	C5—H5A	0.9700
O1—C7	1.396 (3)	C5—H5B	0.9700
O1—C8	1.423 (3)	C6—C7	1.505 (4)
O2—N3	1.250 (2)	C6—H6A	0.9700
O3—N3	1.255 (2)	C6—H6B	0.9700
N1—C3	1.358 (3)	C7—H7	0.9800
N1—C11	1.458 (3)	C8—C9	1.475 (4)
N1—C1	1.475 (3)	C8—H8A	0.9700
N2—C3	1.356 (3)	C8—H8B	0.9700
N2—C7	1.453 (3)	C9—H9A	0.9600
N2—C2	1.457 (3)	C9—H9B	0.9600
N3—C4	1.379 (3)	C9—H9C	0.9600
N4—C14	1.316 (3)	C11—C12	1.497 (3)
N4—C13	1.342 (3)	C11—H11A	0.9700
C1—C2	1.517 (3)	C11—H11B	0.9700
C1—H1A	0.9700	C12—C13	1.377 (3)
C1—H1B	0.9700	C12—C16	1.386 (3)
C2—H2A	0.9700	C13—H13	0.9300
C2—H2B	0.9700	C14—C15	1.369 (3)
C3—C4	1.387 (3)	C15—C16	1.378 (3)
C4—C5	1.491 (3)	C15—H15	0.9300
C5—C6	1.514 (4)	C16—H16	0.9300
			
C7—O1—C8	114.8 (2)	H6A—C6—H6B	108.0
C3—N1—C11	121.70 (18)	O1—C7—N2	112.8 (2)
C3—N1—C1	109.47 (18)	O1—C7—C6	108.2 (2)
C11—N1—C1	117.89 (18)	N2—C7—C6	108.9 (2)
C3—N2—C7	122.79 (19)	O1—C7—H7	108.9
C3—N2—C2	111.33 (18)	N2—C7—H7	108.9
C7—N2—C2	123.86 (19)	C6—C7—H7	108.9
O2—N3—O3	120.5 (2)	O1—C8—C9	109.7 (3)
O2—N3—C4	118.2 (2)	O1—C8—H8A	109.7
O3—N3—C4	121.2 (2)	C9—C8—H8A	109.7
C14—N4—C13	115.5 (2)	O1—C8—H8B	109.7
N1—C1—C2	103.53 (17)	C9—C8—H8B	109.7
N1—C1—H1A	111.1	H8A—C8—H8B	108.2
C2—C1—H1A	111.1	C8—C9—H9A	109.5
N1—C1—H1B	111.1	C8—C9—H9B	109.5
C2—C1—H1B	111.1	H9A—C9—H9B	109.5
H1A—C1—H1B	109.0	C8—C9—H9C	109.5
N2—C2—C1	101.66 (19)	H9A—C9—H9C	109.5
N2—C2—H2A	111.4	H9B—C9—H9C	109.5
C1—C2—H2A	111.4	N1—C11—C12	114.11 (18)
N2—C2—H2B	111.4	N1—C11—H11A	108.7
C1—C2—H2B	111.4	C12—C11—H11A	108.7
H2A—C2—H2B	109.3	N1—C11—H11B	108.7
N2—C3—N1	109.43 (19)	C12—C11—H11B	108.7
N2—C3—C4	120.32 (19)	H11A—C11—H11B	107.6
N1—C3—C4	130.3 (2)	C13—C12—C16	116.7 (2)
N3—C4—C3	122.3 (2)	C13—C12—C11	123.00 (19)
N3—C4—C5	117.1 (2)	C16—C12—C11	120.3 (2)
C3—C4—C5	120.3 (2)	N4—C13—C12	124.8 (2)
C4—C5—C6	111.3 (2)	N4—C13—H13	117.6
C4—C5—H5A	109.4	C12—C13—H13	117.6
C6—C5—H5A	109.4	N4—C14—C15	125.8 (2)
C4—C5—H5B	109.4	N4—C14—Cl	116.08 (18)
C6—C5—H5B	109.4	C15—C14—Cl	118.11 (18)
H5A—C5—H5B	108.0	C14—C15—C16	117.0 (2)
C5—C6—C7	111.6 (2)	C14—C15—H15	121.5
C5—C6—H6A	109.3	C16—C15—H15	121.5
C7—C6—H6A	109.3	C15—C16—C12	120.1 (2)
C5—C6—H6B	109.3	C15—C16—H16	119.9
C7—C6—H6B	109.3	C12—C16—H16	119.9
			
C3—N1—C1—C2	−16.3 (3)	C8—O1—C7—N2	75.1 (3)
C11—N1—C1—C2	128.5 (2)	C8—O1—C7—C6	−164.3 (2)
C3—N2—C2—C1	−19.3 (3)	C3—N2—C7—O1	91.2 (3)
C7—N2—C2—C1	176.5 (2)	C2—N2—C7—O1	−106.4 (3)
N1—C1—C2—N2	20.5 (2)	C3—N2—C7—C6	−28.9 (3)
C7—N2—C3—N1	174.3 (2)	C2—N2—C7—C6	133.5 (2)
C2—N2—C3—N1	9.9 (3)	C5—C6—C7—O1	−67.8 (3)
C7—N2—C3—C4	−6.0 (3)	C5—C6—C7—N2	55.2 (3)
C2—N2—C3—C4	−170.4 (2)	C7—O1—C8—C9	180.0 (2)
C11—N1—C3—N2	−138.6 (2)	C3—N1—C11—C12	−140.6 (2)
C1—N1—C3—N2	4.7 (3)	C1—N1—C11—C12	79.0 (2)
C11—N1—C3—C4	41.7 (4)	N1—C11—C12—C13	23.2 (3)
C1—N1—C3—C4	−175.0 (2)	N1—C11—C12—C16	−159.4 (2)
O2—N3—C4—C3	−179.6 (2)	C14—N4—C13—C12	1.3 (3)
O3—N3—C4—C3	3.1 (3)	C16—C12—C13—N4	−3.6 (3)
O2—N3—C4—C5	7.1 (3)	C11—C12—C13—N4	173.9 (2)
O3—N3—C4—C5	−170.2 (2)	C13—N4—C14—C15	2.3 (3)
N2—C3—C4—N3	−158.8 (2)	C13—N4—C14—Cl	−177.37 (17)
N1—C3—C4—N3	20.8 (4)	N4—C14—C15—C16	−3.2 (3)
N2—C3—C4—C5	14.3 (3)	Cl—C14—C15—C16	176.44 (17)
N1—C3—C4—C5	−166.1 (2)	C14—C15—C16—C12	0.6 (3)
N3—C4—C5—C6	−173.1 (2)	C13—C12—C16—C15	2.4 (3)
C3—C4—C5—C6	13.5 (3)	C11—C12—C16—C15	−175.1 (2)
C4—C5—C6—C7	−48.2 (3)		

### Hydrogen-bond geometry (Å, °)

**Table:**

*D*—H···*A*	*D*—H	H···*A*	*D*···*A*	*D*—H···*A*
C11—H11B···O3	0.97	2.35	2.803 (3)	108
C13—H13···N1	0.93	2.54	2.891 (3)	103
